# Characterization of Mast2 kinase defines structural features, regulation, and substrates

**DOI:** 10.1016/j.jbc.2025.110922

**Published:** 2025-11-17

**Authors:** Michael C. Lemke, Miaomiao Chen, Sophia S. Jang, Michael T. Rader, Nithin R. Avala, Casey J. Bauchle, Rafael Pluguez, Ciara J. Miller, John D. Butler, Stefan R. Hargett, Daniel S. Lank, Brandon D. Seltzer, Daniel S. Stornetta, Zheng Fu, Ku-Lung Hsu, Thurl E. Harris

**Affiliations:** 1 Department of Pharmacology, University of Virginia School of Medicine, Charlottesville, Virginia, USA; 2 Department of Chemistry, University of Virginia, Charlottesville, Virginia, USA; 3 Department of Biology, University of Virginia, Charlottesville, Virginia, USA; 4 Department of Chemistry, University of Texas at Austin, Austin, Texas, USA

**Keywords:** kinase, AGC, phosphorylation, MAST2, MAST1, MAST3, MAST4, mTOR, signal transduction, ENSA, DUF 1908, AlphaFold

## Abstract

The mammalian microtubule-associated serine/threonine (MAST) kinases are a highly conserved subfamily of AGC kinases that are implicated as therapeutic targets for cancer and diabetes. However, the activity, regulation, and substrates of MAST kinases are poorly understood. We examined the biochemical activity of Mast2, as a representative of the MAST family. The domain of unknown function (DUF1908) is necessary for Mast2 kinase activity *in vitro*, while the PDZ domain is dispensable. Mast2 kinase activity does not appear to be compatible with the AGC kinase model of T-loop phospho-activation. Instead, it contains a unique insertion that is likely stabilized by ion-pair interactions. The C terminus of the kinase domain contains motifs regulated by mechanistic target of rapamycin (mTOR) in other AGC kinases, and mutation of these conserved residues reduces Mast2 kinase activity. Consistent with mTOR regulation, Mast2 purified from insulin-stimulated cells has increased activity compared to serum-starved cells, and this increase in activity is dependent on mTOR. Finally, stable ^18^O-ATP labeled kinase assay linked phospho-proteomics identifies a collection of putative Mast2 substrates, including the PP2A inhibitor, endosulfine-α. Our results develop a biochemical profile of the MAST kinases, provide insight into their regulatory mechanisms, and begin to identify the cellular function of MAST2.

Eukaryotic protein kinases are essential enzymes that phosphorylate targeted downstream proteins. Historically, protein kinases have been promising pharmacological targets within the druggable proteome, given their role in integrating numerous critical cellular processes, including cell survival and metabolism (1, 2). Although the structure, activation, regulation, and substrate specificity of numerous kinases are thoroughly characterized, the microtubule-associated serine/threonine (MAST) family of kinases remains underexplored (3, 4, 5, 6). MASTs are well-conserved across eukaryotes but are not present in bacteria (7). MAST kinase catalytic cores are highly homologous to those of protein kinases A, G, and C (AGC kinases) (8). Humans express five distinct MAST kinases throughout the body, MAST1-4 and the MAST-like kinase, MASTL (4, 9, 10). All five MAST kinases have been associated with various pathologies that primarily cluster into two categories: cancer (11, 12, 13, 14, 15), and abnormal development (4, 14, 16, 17, 18, 19).

MASTs are unique within the AGC kinase superfamily due to their multidomain structure with extensive interspersed and intrinsically disordered sequences (8, 10). MAST1-4 contain three domains: a domain of unknown function (DUF1908, referred to as DUF hereafter), the AGC kinase domain, and a post synaptic density protein (PSD95), *Drosophila* disc large tumor suppressor (Dlg1), and zonula occludens-1 protein (zo-1) (PDZ) domain (4). MASTL differs in that it only has a divided AGC kinase domain (20). MASTL is the most well-characterized MAST with a known function, an oncogene activated by CDK1 to regulate mitotic entry through the protein phosphatase 2A (PP2A) inhibitor molecule endosulfine-α (ENSA) (20, 21, 22, 23, 24). AGC kinases contain an activation segment, or T-loop, a short unstructured sequence of residues spanning from the canonical Asp-Phe-Gly (DFG) to Ala-Pro-Glu (APE) motif in the catalytic domain (20, 25). The MAST kinases are unique in the AGC family as each contains a T-loop insertion that interrupts the traditional location of the activation loop phosphosite (APE_-9_ (26)). MASTL has a T-loop insertion that spans hundreds of amino acids and is stimulated by multiple phosphorylation events (20, 24, 27, 28). The insertions in MAST1-4 are just 15 amino acids long. It is unknown whether MAST1-4 are activated through T-loop phosphorylation. However, studies in *Caenorhabditis elegans*, which lack a MASTL homolog but have a single MAST homolog (kin-4) that includes DUF, kinase, and PDZ domains, show that kin-4 can functionally replace MASTL through direct phosphorylation of MASTL substrates. These studies also reveal that MAST kinases lack a phosphosite in the correct position for traditional AGC T-loop activation (24).

Full activation of most AGC kinases requires phosphorylation of the T-loop as well as a pair of highly conserved carboxy-terminal regulatory motifs, the turn motif (TM), and the hydrophobic motif (HM), frequently regulated by the mTOR complexes (mTORC1/2) (8, 29, 30). Phosphorylation of the HM is necessary to promote stabilization of the kinase in the active conformation, while TM phosphorylation aids in positioning the HM and enhancing stability (8). The TMs and HMs of AGC kinases are often targets of mTORC2; however, S6K is targeted by mTORC1 (8). A kinome-wide RNAi screen parallel with tuberous sclerosis 2 protein (TSC2) knockdown identified the *Drosophila melanogaster* MAST homolog (drop-out, dop) as an effector kinase required for mTORC1-mediated growth stimulated by TSC2 deficiency (31). A different RNAi screen in human cells also identified MAST2 and MASTL as positive regulators of insulin/IGF-1-stimulated GLUT4 translocation to the plasma membrane (32). Proteomic screens of rapamycin-sensitive proteins have identified phosphosites in MAST2 (33). Finally, MASTL activity has been demonstrated to be regulated by mTORC1 *via* phosphorylation (34). These results suggest that the other MASTs, like MASTL, may be downstream effectors of mTOR.

Here, we present our efforts to develop a biochemical platform for purified recombinant mammalian MAST1-4 kinases, primarily Mast2, as assessed by activity *in vitro*. Using this approach, we investigated the contribution of each conserved domain and residues in conserved motifs to gain an understanding of the catalytic requirements and regulatory inputs of the remaining MAST kinases. Finally, using a modified and untargeted stable-isotope kinase-assay-linked phosphoproteomic (SIKALIP) (35) method, we identify putative target proteins in HEK293T cells to establish the cellular role of Mast2. Considering how the MAST kinase field is still in the early stages of investigation compared to other kinase families, our work provides the biochemical foundation for future system-wide functional mapping and clinical studies to guide therapy development.

## Results

### Purification and enzymatic activity of recombinant Mast kinases

**Figure 1:**
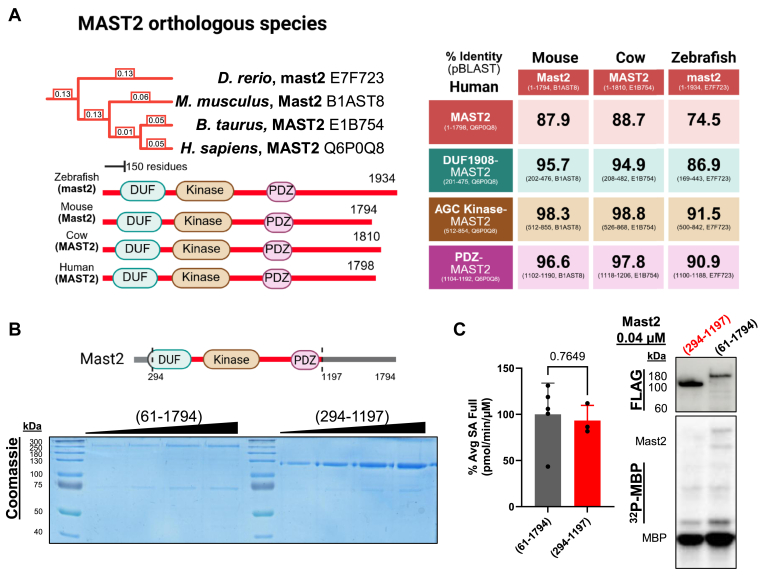
**Recombinant Mast2 61 to 1794 and 294 to 1197 have similar activity.***A*, Clustal Omega phylogeny and node branch length of selected ortholog species that conserve MAST2, visualized using the iTOL webserver (104) (*Danio rerio*, E7F723; *Mus musculus*, B1AST8; *Bos taurus*, E1B754; and *Homo sapiens*, Q6P0Q8). Percent identities comparing whole protein sequences and conserved domains of Mast2 as analyzed by multiple sequence alignment in NIH protein-BLAST (pBLAST % Identity). *B*, diagram of Mast2 and Mast2 (294–1197). Recombinant Mast2 was affinity-purified from HEK293T cells and eluted with FLAG peptide. SDS-PAGE resolution and Coomassie blue visualization of anti-FLAG affinity-purified recombinant lengthened (61–1794) and truncated Mast2 (294–1197). *C*, representative autoradiogram and FLAG immunoblot for the average specific activity calculated per molar concentration of kinase (pmol/minute/μM) of both recombinant (61–1794) and (294–1197) Mast2 on 10 μg of MBP as substrate, normalized to the maximal amount of activity measured for Mast2 (61–1794). Statistical test is unpaired two-tailed *t* test. Graphics were created in BioRender. Lemke, M. (2025) https://BioRender.com/w79m744. MAST, microtubule-associated serine/threonine; MBP, myelin basic protein; NIH, National Institutes of Health.

The MAST kinase family comprises large tri-domain kinases with intrinsically disordered sequences at the N and C termini (10) (Fig. 1*A*). The regions containing the DUF, AGC kinase, and PDZ domains are highly conserved in chordates (with ∼87–99% identity, Fig. 1*A*). The N and C termini of the complete paralog sequences are more variable between species (whole sequences range from ∼75–88% identity, Fig. 1*A*). To examine kinase activity, we purified Mast2 from human cells. HEK293T cells were transiently transfected with a construct expressing an amino-terminal FLAG-tagged Mast2 (61–1794) and purified *via* FLAG affinity chromatography (Fig. 1*B*). Due to the exceptionally low yield of this construct, together with the sequence variability of both termini in various MAST proteins, led us to decide to produce a terminally truncated version of Mast2 that retains the tri-domain architecture, Mast2 (294–1197). Equal molar amounts of Mast2 (61–1794) and (294–1197) were incubated with [γ-^32^P]-ATP and myelin basic protein (MBP) as an artificial substrate. The activity of Mast2 (294–1197) was the same as Mast2 (61–1794) (Fig. 1*C*), suggesting that the nonconserved regions at the N and C termini are not essential in an *in vitro* kinase assay. Interestingly, we observed that both (61–1794) and truncated Mast2 coimmunoprecipitated from HEK293T cells with a protein of approximately 70 to 75 kDa, which had been previously described (36). Liquid chromatography tandem mass spectrometry (LC-MS/MS) identified HSP70 as the most abundant accompanying protein. AlphaFold 3 predicts HSP70 to interact with MAST2 in the intrinsically disordered region between the catalytic and PDZ domains (Fig. S1).

### Enzymatic determinants of Mast2 kinase activity

**Figure 2:**
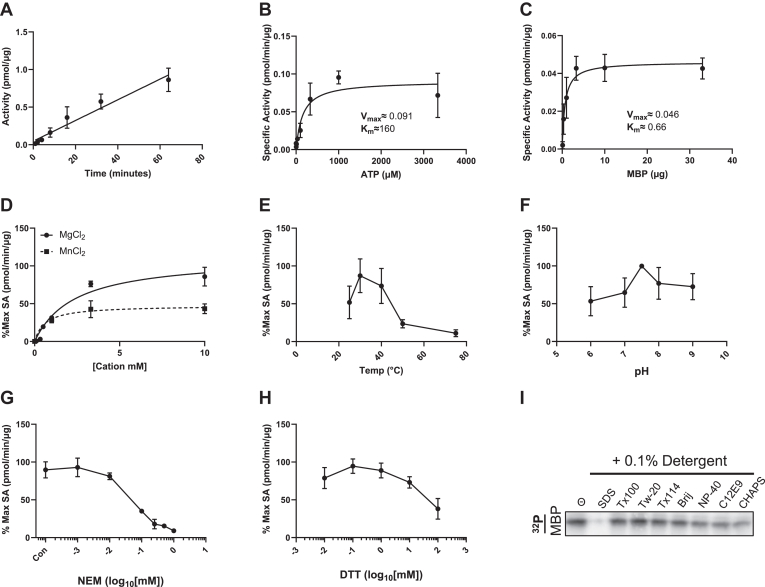
**The enzymatic profile of Mast2 (294–1197).** Recombinant Mast2 (294–1197) was affinity-purified from HEK293T cells and eluted with FLAG peptide. *A*, Mast2 (294–1197) kinase activity using 10 μg MBP for 1, 2, 4, 8, 16, 32, and 64 min. *B*, Mast2 kinase activity with 1, 3.3, 10, 33, 100, 333, 1000, and 3333 μM ^32^P-ATP. *C*, Mast2 kinase activity with 0.1, 0.33, 1, 3.3, 10, and 33 μg of MBP. *D*, Mast2 (294–1197) kinase activity with ascending concentrations of MgCl_2_ or MnCl_2_ (*solid line* and *dotted line*, respectively). 0, 0.1, 0.33, 0.5, 1, 3.33, and 10 mM final reaction concentration for Mg, and 0, 1, 3.33, and 10 mM final reaction concentration for Mn. *E*, Mast2 (294–1197) kinase activity with 10 μg of MBP at 25, 30, 40, 50, and 75 °C. *F*, Mast2 (294–1197) preincubated in 1 mM bis-tris-propane (BTP) kinase buffer (pH range 6–9) for 15 min before kinase reaction. *G*, Mast2 (294–1197) preincubated in ascending amounts of N-ethylmaleimide (NEM) 15 min before the kinase reaction. *H*, Mast2 (294–1197) preincubated in ascending amounts of dithiothreitol (DTT) 15 min before the kinase reaction. *I*, Mast2 (294–1197), was preincubated without (⊝ Control) or with 0.1% volume of SDS, Triton X-100 (T x 100), Tween-20 (Tw-20), Triton X-114 (T x 114), Brij-35 (Brij), NP-40, C12E9, and CHAPS surfactants before the kinase reaction with (294–1197). V_max_ and K_m_ in *B* and *C* are calculated using the Michaelis-Menten nonlinear fit model in GraphPad Prism. MAST, microtubule-associated serine/threonine; MBP, myelin basic protein.

We proceeded with our truncated version to evaluate the *in vitro* reaction constraints. Mast2 activity was linear up to 60 min at 30 °C (Fig. 2*A*). Next, we calculated the substrate kinetics for ATP (K_m_ ≈ 77–300 μM (95% confidence interval [CI]), Fig. 2*B*) and a generic substrate, MBP (K_m_ ≈ 0.43–0.99 μg (95% CI), Fig. 2*C*). Magnesium or manganese availability mediates kinase binding to the γ-phosphate of ATP binding *via* the conserved Asp-Phe-Gly (DFG) motif in the active site cleft of protein kinases, as well as other ion-pair residues in the N-lobe (8). In our assay, Mast2 is maximally stimulated by Mg^2+^ rather than Mn^2+^ (Fig. 2*D*). Mast2 also demonstrated highest activity at 30 °C (Fig. 2*E*) and a pH of 7.5 (Fig. 2*F*). To assess the contribution of cysteine residues to Mast2 kinase activity, we assayed Mast2 with N-ethylmaleimide or DTT. Mast2 demonstrates sensitivity to alkylation and reduction, highlighting the contribution of unbound cysteines to kinase activity (Fig. 2, *G* and *H*). Finally, we assessed the sensitivity of Mast2 kinase activity to different detergents during the kinase reaction. We found that Mast2 demonstrated vulnerability to charged detergents, as it was inactivated by the anionic detergent SDS and the zwitterionic detergent CHAPS (Fig. 2*I*). Sensitivity to CHAPS may indicate that Mast2 is hypersensitive to changes in surface charge, or that kinase activity is dependent on subtle or reversible assembly, intramolecular interactions, or thermal stability without complete enzyme denaturation (37).

### MAST kinase orthologs

**Figure 3:**
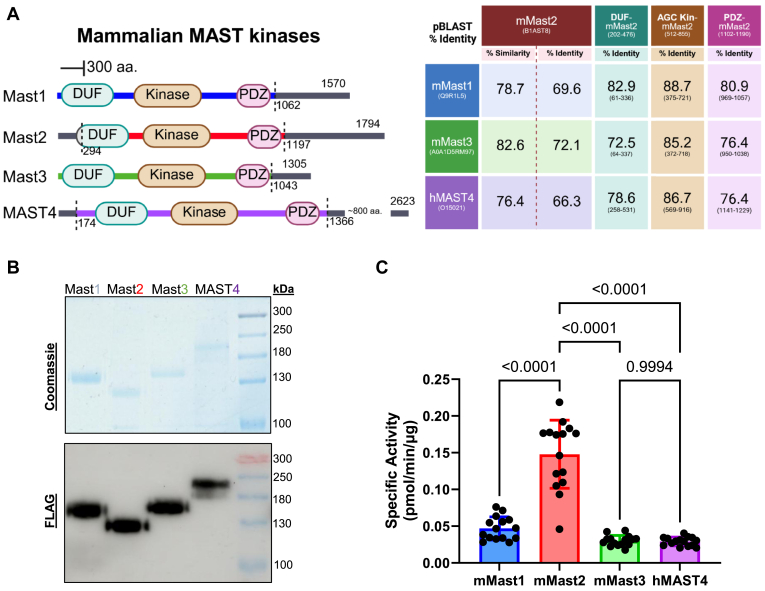
**Purification and kinase activity of truncated MAST1-4.** HEK293T cells were transfected with plasmid constructs expressing truncated mammalian MAST1-4. *A*, diagram representation of the numbered Mast isoforms. Truncations for Mast1 (1–1062), Mast2 (294–1197), Mast3 (1–1043), and MAST4 (174–1366) are outlined by the *dashed line* in the sequence. Percent identity of Mast1-3 (Q9R1L5, B1AST8, and A0A1D5RM*97*, respectively) and MAST4 (O15021) purified as recombinant proteins. *B*, FLAG-affinity purified Mast1 (1–1062), Mast2 (294–1197), Mast3 (1–1043), and MAST4 (174–1366) proteins were resolved by SDS-PAGE and visualized by Coomassie brilliant blue (*top*) or FLAG immunoblot (*bottom*). *C*, comparative kinase activity of purified Mast kinases represented as a function of protein amount (pmol/min/μg). Statistical test is one-way analysis of variance (ANOVA) with Tukey's multiple comparison *post hoc*. Graphics were created in BioRender. Lemke, M. (2025) https://BioRender.com/w13j433. MAST, microtubule-associated serine/threonine.

The MAST2 paralog isoforms are strikingly similar when comparing conserved domains (Fig. 1*A*). By comparison, MAST1-4 kinase paralogs also conserve the DUF1908, kinase, and PDZ domains, and the mouse Mast1-3 and human MAST4 share a high degree of domain identity (range 72–89% identity (Fig. 3*A*)). The sequence lengths of MAST1-4 isoforms are disparate, the shortest often being MAST3 (1309 aa. in humans, O60307) and the longest being MAST4 (2623 aa. in humans, O15021). To assess if our assay could be used to test the activity of MAST kinases, we measured the kinase activity of all the mammalian MAST kinases by expressing and purifying truncated isoforms of mammalian MAST1-4 (Fig. 3*B*). FLAG-tagged mouse Mast1 (1–1062), mouse Mast3 (1–1043), and human Mast4 (174–1366) were transiently expressed in HEK293T, and affinity purified as described for Mast2 (294–1197) (Fig. 3*B*). We found that the activity of the other truncated Mast kinases was within a similar range of specific activities. Mast2 had the highest activity, and Mast3/4 had the lowest on MBP (Fig. 3*C*). This demonstrates that all four Mast kinase isoforms are functional Ser/Thr kinases *in vitro* and can tolerate terminal truncations.

### Identifying a functional role of the DUF1908 domain

**Figure 4:**
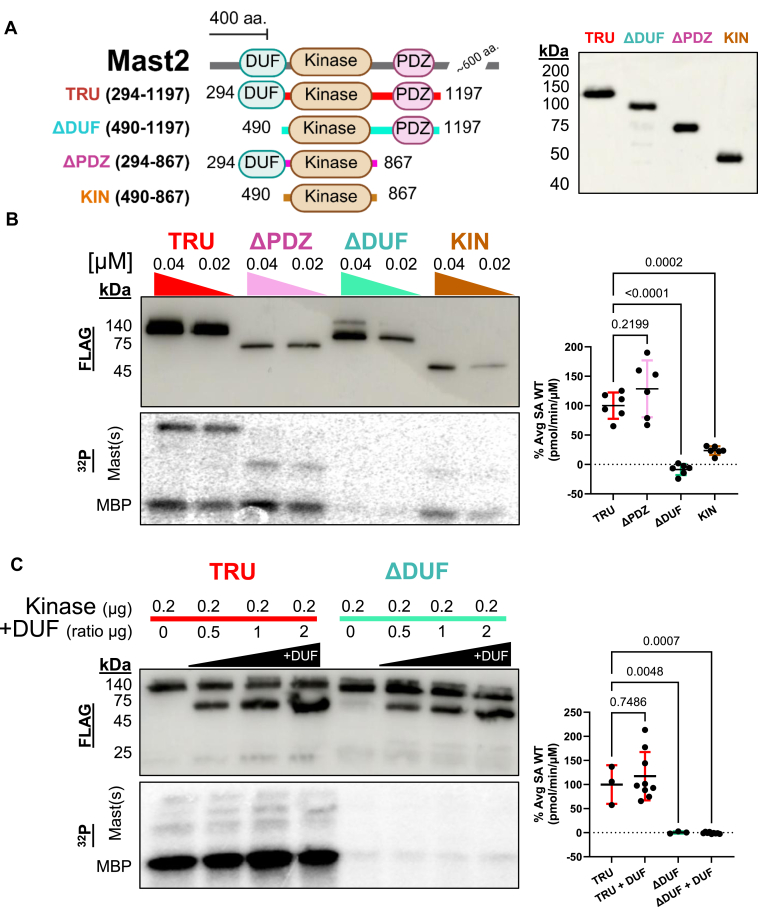
**The DUF domain is necessary for Mast2 (294–1197) activity.** Truncation mutants of Mast2 (294–1197, TRU). *A*, graphical visualization of the Mast2 protein sequence with conserved protein domains (DUF1908, KIN, and PDZ in order) and corresponding truncation constructs (Mast2 (490–1197) ΔDUF-*cyan*, Mast2 (294–867) ΔPDZ-*pink*, and Mast2 (490–867) KIN-*orange*) (*left*). FLAG immunoblot of the N-terminal FLAG-tagged recombinant Mast2 constructs and truncation mutants, Mast2 (294–1197), (490–1197), (294–867), and (490–867) (abbreviated TRU, ΔDUF, ΔPDZ and KIN) (*right*). *B*, representative FLAG western blot (*left-top*) and autoradiogram (*left-bottom*) of comparative specific activities of equimolar concentrations of purified Mast2 TRU, ΔDUF, ΔPDZ, and KIN on MBP. Comparative specific activities were quantified per molecule of kinase (pmol/min/μM, right). *C*, representative FLAG western blot (*left-top*) and autoradiogram (*left-bottom*) of comparative specific activities of purified Mast2 TRU and ΔDUF with increasing ratios of purified Mast2 (61–495, DUF) to μg of Mast2 (294–1197) and (490–1197) on MBP quantified per molecule of kinase (*right*). Statistical test is one-way ANOVA with Dunnett's multiple comparison *post hoc*. Graphics were created in BioRender. Lemke, M. (2025) https://BioRender.com/q54j083. MAST, microtubule-associated serine/threonine; MBP, myelin basic protein.

We next assessed the contribution of each conserved domain for mammalian recombinant Mast2 kinase activity (Fig. 4*A*). We generated N-terminal FLAG-tagged constructs lacking either the DUF1908 domain (ΔDUF, 490–1197), the PDZ domain (ΔPDZ, 294–867), or containing only the AGC homologous catalytic core (KIN, 490–867). We find that both the ΔDUF and KIN had significantly less kinase activity than Mast2 (294–1197, TRU) or ΔPDZ (Fig. 4*B*). This demonstrates that although the PDZ domain is dispensable for an *in vitro* kinase reaction on an artificial substrate, the DUF domain is necessary for catalytic activity. To test if the sequence comprising the DUF domain of Mast2 (61–495, DUF) might be sufficient to rescue the inactivity of the ΔDUF, we supplemented Mast2 TRU and Mast2 ΔDUF with recombinant purified DUF *in trans*. However, the addition of the DUF did not affect TRU kinase activity, nor was it able to rescue ΔDUF inactivation (Fig. 4*C*). It is therefore likely that the removal of the DUF domain disrupts critical intramolecular interactions and folding that could not be mimicked by supplying in *trans*.

### Irregularity of Mast2's activation loop

**Figure 5:**
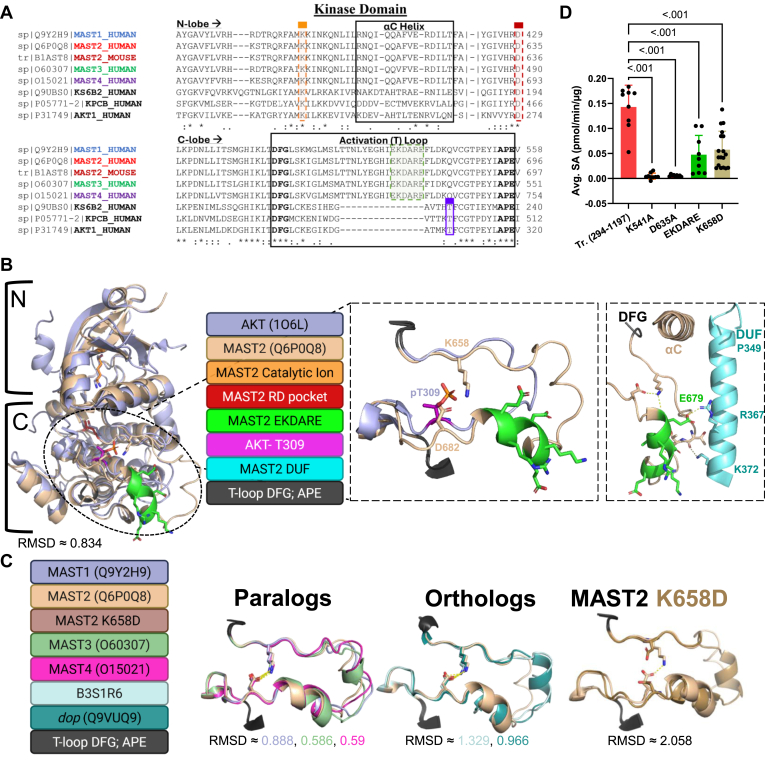
**MAST kinases have unique T-loop interactions.** Point mutations in the Mast2 kinase domain. *A*, multiple sequence alignment featuring sections of Mast kinase domains compared to the catalytic core of other AGC kinase family members (Akt, S6K, and PKC) created in Clustal Omega. Conserved residue and motif numbers are based on Uniprot sequences within catalytic and regulatory regions of mouse and human MAST kinases (∗ = identical; : = high conservation; . = low conservation). *B*, AlphaFold visualization of the MAST2 (Q6P0Q8-tan) kinase domain over the solved kinase domain of human AKT (1O6L-*blue*, *left*), both kinase activation loops (*middle*), and the putative interdomain DUF to T-loop contact site in MAST2 (*left*). Boundaries of the putative N (*small*) and C (*large*) lobes of the kinase domain are outlined. Numbers indicate amino acids. *C*, AlphaFold visualization of the DFG-APE sequences in MAST paralogs MAST1 (Q9Y2H9), MAST2 (Q6P0Q8), MAST3 (O60307), MAST4 (O15021); MAST orthologs *T. adhaerens* (B3S1R6), and *dop* (Q9VUQ9); and MAST K658D (AlphaFold 3, (105)). RMSD was calculated in Pymol modeling (Schrödinger). *D*, comparative average specific activity (pmol/min/μg) of purified Mast2 (294–1197) with kinase domain point mutations (K541-*orange*, D635-*maroon*, EKDARE-*green*, K658D-*light brown*). Statistical test is an one-way ANOVA with Dunnett's multiple comparison *post hoc*. Graphics were created in BioRender. Lemke, M. (2025) https://BioRender.com/38spi2k. MAST, microtubule-associated serine/threonine.

The activation or T-loop is a structural part of kinase mechanics that also helps distinguish one T-loop from others in upstream kinases and establishes a significant level of substrate specificity for the kinase itself. (38). We currently understand kinase activation, or T loops, as regulated by the disordered T loop, or DFG^out^ conformation, that blocks the active site. Phosphorylation of the loop at a conserved site facilitates alignment with other catalytic components, such as the αC helix, to form the active, DFG^in^ conformation (8, 39, 40). The T-loops of MAST kinases differ from other kinases in the AGC superfamily by two key features: first, the embedded insertion, as mentioned ranging from 15 amino acids for MAST1-4 and over 500 for MASTL; second, the absence of the conserved phosphosite at APE^−9^ (S6k-T229, Akt-T308, Fig. 5*A*/Fig. 5*B*). MASTL is the only MAST kinase with an experimentally solved structure that excludes its insertion (5LOH and 8V5H (41)). Viewing the linear sequences and predicted models of MAST kinases demonstrates conservation of canonically critical residues throughout the N and C lobes of the kinase fold. The MAST kinases conserve residues that bind ATP (K541-catalytic ion) and cation-ATP conjugates (D635 human—RD pocket) (Fig. 5, *A* and *B*) (8). Although only a prediction, the MAST2 kinase domain model (Q6P0Q8) also reveals two additional interesting T-loop features: a highly conserved internal ion-pair interaction between K658 (DFG^+3^) and D682 (APE^-11^) that has been postulated to replace the loop phosphosite in the *Caenorhabditis elegans* MAST homolog (24) (Fig. 5*B*/Fig. 5*C*), and a putative DUF domain: T-loop interface (Fig. 5*B*). This latter interaction is predicted to occur between an alpha-helix in the DUF domain and a polar region of the MAST-specific insertion of the T-loop.

To evaluate the contribution of the MAST-unique T-loop features, we first determined the range of Mast2 (294–1197) activity by performing alanine missense mutations on either K541 or D635 catalytic residues, which eliminated most of Mast2's kinase activity (Fig. 5*D*). The DUF: T-loop interface within the AlphaFold MAST2 model was stabilized by polar side chain contacts involving the latter half of the T-loop insertion, the EKDARE motif, hereafter. Mutating the EKDARE motif to AAAAAA caused a threefold decrease in kinase activity (Fig. 5*D*). This result, supported by kinase inactivation in the DUF truncation experiment (Fig. 4), is consistent with the T-loop of Mast2 (294–1197) as stabilized *in cis* by the DUF domain *in vitro*. No activating phosphosites in the T-loops of MAST1-4 have been identified. To explore the functional effects of changing the proposed MAST T-loop ion-pair, we tested the *in vitro* activity of MAST2 K658D. We hypothesized that disrupting this ion pair would clash with the natural basal aspartate in the lower part of the T-loop, thereby destabilizing it. This was supported by the increased structural deviation seen in the model of MAST2 K658D compared to wild type (Fig. 5*C*). Mutation of K658D impairs Mast2 activity as it exhibited about one-third of the activity compared to wild type on an artificial substrate. Finally, we should point out that R634 in the HRD motif, which in other AGC kinases typically forms an ion pair with the activation loop phospho-Thr, is shown by the model to interact ionically with D682 within the activation loop. This may also provide stabilization to the activation loop.

### Regulation of Mast2's C-terminal kinase domain

**Figure 6:**
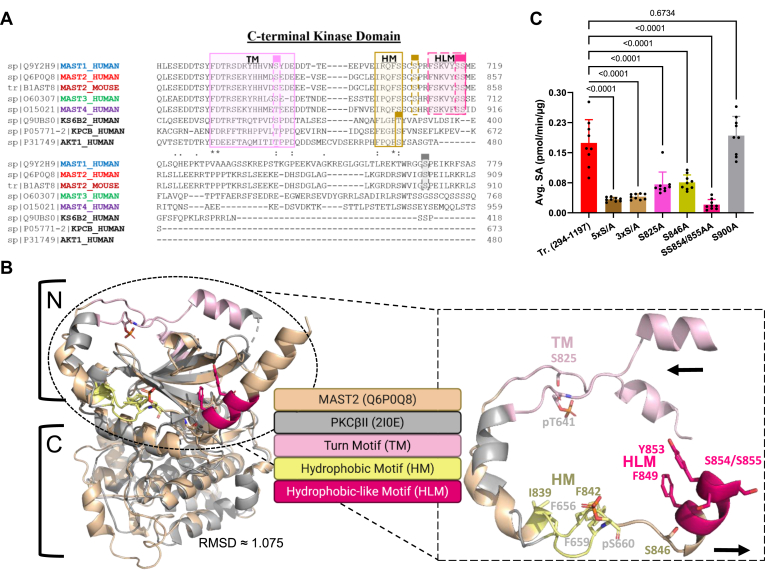
**Mast2 activity is sensitive to point mutations in conserved AGC kinase regulatory motifs.***A*, multiple sequence alignment featuring sections of Mast kinase domains with the C-terminal kinase domain extensions of other AGC kinase family members (Akt, S6K, and PKC) created in Clustal Omega. Conserved residue and motif numbers are based on Uniprot sequences within catalytic and regulatory regions of mouse and human MAST kinases. *B*, AlphaFold visualization with corresponding MAST2 (Q6P0Q8-tan) kinase domain (*left*) and its activation loop (*right*) superimposed over the solved structure for human PKCβII (2I0E-*light gray*). Numbers indicate amino acids. Boundaries of the putative N (*small*) and C (*large*) lobes of the kinase domain are outlined. RMSD was calculated in PyMOL modeling (Schrödinger). *C*, comparative average kinase specific activity (pmol/min/μg) of purified Mast2 (294–1197) with AGC kinase C-terminal motif point mutations individually (S825A-*light pink*, S846A-*gold*, SS854/855AA-*dark pink*, S900A-*gray*) as well as in aggregate (5 × S/A = S825A + S846A + SS854/855AA + S900A-*dark brown*; 3 × S/A = S846A + SS854/855AA-*light brown*). Statistical test is one-way ANOVA with Dunnett's multiple comparison *post hoc*. Graphics were created in BioRender. Lemke, M. (2025) https://BioRender.com/38spi2k. MAST, microtubule-associated serine/threonine.

In AGC kinases, the sequence immediately after the kinase domain operates as a C-terminal phosphoregulatory tail by influencing the kinase's catalytic activity and allosteric regulation, which is typically targeted by mTOR (5, 9, 42). The canonical TM and HM are phosphorylated within the C-terminal tail to facilitate better hydrophobic interactions between the Phe (F) residues of these motifs with hydrophobic portions of the N-lobe, thus establishing the fully functional regulatory spine (Fig. 6*A*). The structure of the fully phospho-activated AGC kinase PKC (8, 43) demonstrates the role phosphorylation plays in enabling active conformation. Superimposing the structure of MAST2 (Q6P0Q8) with PKCβII (2I0E) highlights both the degree of conservation and divergence of the TM and HM structures between AGC kinase C-terminal extensions (Fig. 6*B*). The C terminal extensions of many AGC kinase domains end just after the hydrophobic motif. Some subsets of AGC kinases that are mTOR-dependent, and specific families like MAST kinases, feature further extended C termini often associated with additional regulatory functions (44). MASTL conserves and autophosphorylates its TM, but alanine mutants retain functionality in cell culture (45). MASTL does not have an HM, relying on hydrophobic interactions *in trans* (45).

All four mammalian MAST kinases conserve a TM phosphosite, with each found to be phosphorylated multiple times in large-scale mass spectrometry phosphoproteomic screens (Fig. 6*A*: MAST1 (S687), MAST2 (S825), MAST3 (S680), and MAST4 (T883)) (46). The HM in AGC kinases is defined by the sequence FxxFS, where the serine is a key mTOR-regulated phosphorylation site crucial for kinase activation. In contrast, MAST kinases contain a divergent HM sequence: IxxFS. Notably, the serine in this motif has not been reported as phosphorylated (PhosphoSitePlus (46)). However, phosphorylation has been detected at the HM Ser^+3^ position in MAST1 (S708) and MAST2 (S846), suggesting conserved regulatory phosphorylation near the HM region despite sequence divergence (46). This indicates that while MAST kinases may lack the canonical HM phosphorylation site, they may retain phosphorylation-dependent regulation adapted to their unique HM motif. The C termini of MAST kinases then extend into what we have termed a hydrophobic-like motif (HLM), with the sequence of FxKV^Y^/_F_SS. Both Ser residues in the HLM of human MAST2 (SS854/855) and MAST3 (SS709/710) have been reported to be phosphorylated, whereas only one phospho-Ser has been seen in the HLM of MAST1 (S717) and MAST4 (S914) (46).

We hypothesized that the conserved C-terminal phosphosites in and around the TM, HM, and HLM of Mast2 (294–1197) regulate kinase activity. Aggregate alanine mutations of five serine residues within the TM (S825A), HM (S846A), HLM (SS854/855), and S900, a previously identified rapamycin-sensitive site in a large-scale screen (33), reduced activity by fivefold (5 × S/A, Fig. 6*C*). Combining just S846 and SS854/855 resulted in a similar reduction (3 × S/A, Fig. 6*C*). Individual motif mutations S825A, S846A, or SS854/855AA also significantly decreased *in vitro* activity, underscoring the importance of each of these motifs in MAST kinases (Fig. 6*C*). Mutation of Ser 900 to Ala did not affect kinase activity. Our findings collectively highlight the catalytic importance of the conserved TM and HM sites, as well as the MAST-specific HLM serine pair.

### Mast2 is stimulated by insulin-mTORC1 independent of the turn motif

**Figure 7:**
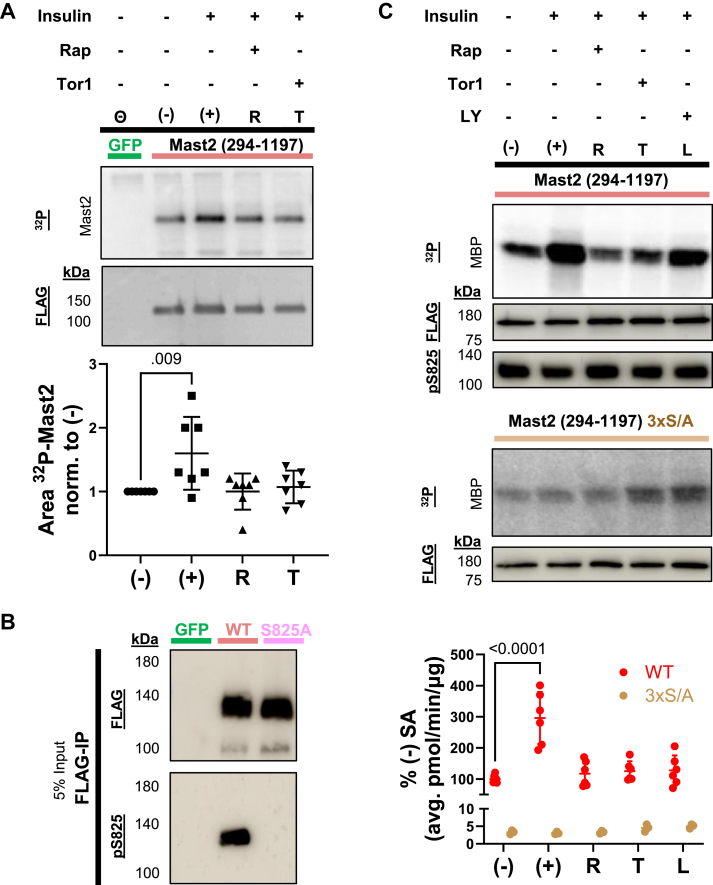
**mTOR inhibition prevents insulin-Mast2 stimulation, regardless of turn motif phosphorylation.** Mast2 (294–1197) was purified from mTOR inhibitor (mTORi) treated cells. *A*, representative autoradiogram (*left-top*) and FLAG-immunoblot (*left*-*bottom*) of the pull-down of Mast2 (294–1197) and GFP from HEK293 cells. Cells were pretreated for 3 to 4 h with Insulin (0.07 or 0.7 μM) and mTORi rapamycin or Torin1 (R and T, 0.25 or 1 μM) with the addition of 0.2 mCi ^32^P-orthophosphate before lysis and FLAG-immunoprecipitation. Bead fractions were eluted with Laemmli buffer and loaded into SDS-PAGE for autoradiography and subsequent FLAG-immunoblot visualized as average quantification of AU (bottom) from labeled Mast2 relative to Insulin-only control. Statistical test is one-way ANOVA with Dunnett's multiple comparison *post hoc*. *B,* efficacy of novel antibodies generated against pS825-MAST2 was confirmed by immunoblot of purifications of Mast2 (294–1197) and Mast2 (294–1197) S825A constructs. *C*, HEK293E cells overexpressing Mast2 (294–1197) or Mast2 (294–1197) 3 × S/A were starved of serum overnight and treated with 250 nM of Rapamycin (R), Torin1 (T), Wortmannin (W), or LY29402 (L) at 10 μM 1 h before acute insulin administration at 1 μM for 15 min. The activity of eluted protein purifications was tested by radiolabeled substrate assay on MBP (^32^P) and quantified as a part of the average percent specific activity (pmol/min/μg) of insulin-stimulated (+) Mast2 (294–1197). Representative autoradiograms and corresponding FLAG-immunoblot of Mast2 (294–1197) and 3 × S/A aggregate mutant construct are shown, along with pS825-immunoblot of Mast2 (294–1197) purifications. Statistical test is two-way ANOVA with Dunnett's multiple comparison *post hoc*. MAST, microtubule-associated serine/threonine; MBP, myelin basic protein.

Many AGC kinases, including AKT, S6K, and some PKC isoforms, are regulated in their C-terminal tails by mTOR kinase complexes (mTORC1/2) in response to insulin (8, 30). Because its C-terminal tail is sensitive to mutations, we next aimed to investigate whether Mast2 (294–1197) is regulated by insulin-mTOR. When isolating Mast2 from cells radiolabeled with ^32^P-orthophosphate, treated with or without insulin and mTOR inhibitors such as rapamycin and Torin1, we found that rapamycin alone was sufficient to block the slight increase in insulin-stimulated overall phosphorylation (Fig. 7*A*).

Considering the high conservation of the TM and reported phosphorylation of the site in all four MAST kinases, we developed a novel phospho-specific antibody targeting the TM (pS825, Fig. 7*B*). Insulin stimulation led to a threefold increase in Mast2 (294–1197) kinase activity *in vitro* (Fig. 7*C*). However, the enhanced kinase activity of Mast2 purified from insulin-treated cells was prevented when cells were pretreated with rapamycin (mTORC1), Torin1, (pan-mTOR), and LY294002 (PI3K) inhibitors (Fig. 7*C*). The pS825 status of the Mast2 (294–1197) was unchanged by insulin stimulation or mTOR inhibition, indicating the TM phosphosite is not regulated by mTORC1 or mTORC2 (Fig. 7*C*). We next examined the HM and HLM domain sensitivity to insulin stimulation. Purified Mast2 (294–1197) 3 × S/A, which is wild type for S825 but includes S846A and SS854/855AA mutants, was not stimulated by insulin (Fig. 7*C*). Overall, our data indicate that Mast2 activity is enhanced by insulin through direct or indirect phosphorylation events that are independent of the TM, require the availability of S846 and SS854/855, and is sensitive to inhibition of mTORC1.

### Substrate screen identifies Mast2 target proteins

**Figure 8:**
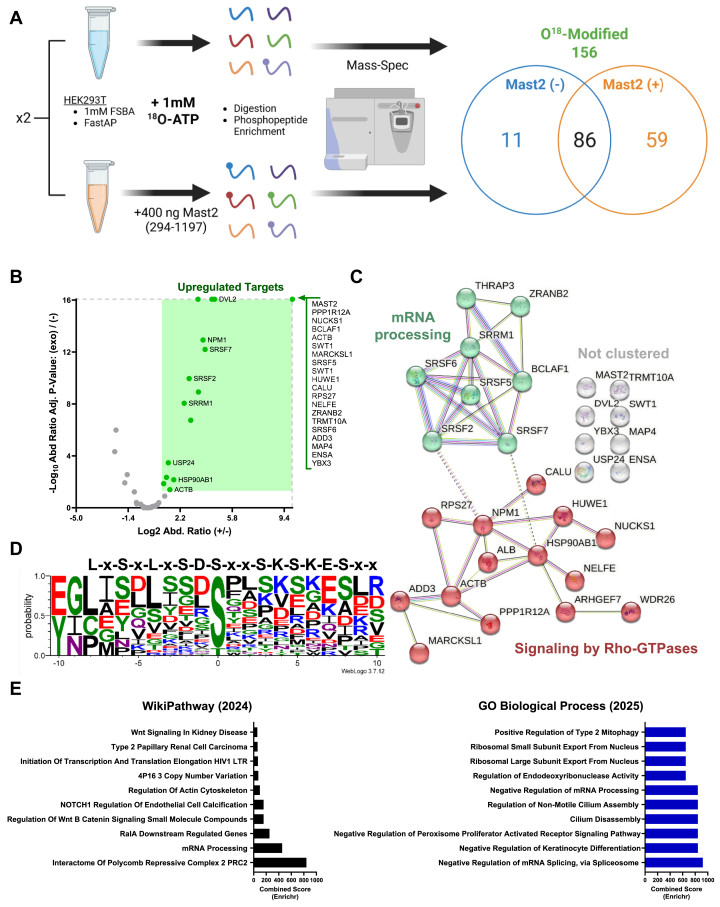
**Mast2 SIKALIP substrate screen.** Mast2 (294–1197) substrate assay. *A*, SIKALIP workflow. Two independent preparations of HEK293T lysates were generated and incubated with FSBA, FastAP, and washed before an *in vitro* kinase reaction with (+) or without (−) exogenous Mast2 (294–1197) in the presence of 1 mM ^18^O-ATP (Cambridge Isotope Laboratories). Once quenched, each reaction was enzymatically digested and enriched for phosphopeptides by TiO_2_. Peptides were analyzed *via* LC-MS/MS using the Proteome Discoverer data pipeline (3.1.0.638, Thermo). The Venn diagram outlines the pooled ^18^O-modified peptide groups corresponding to (+) and (−) sample preparations. *B*, volcano plot representation of ^18^O-modified peptide groups. Exported peptide groups with Byonic score ≥ 200 were graphed according to their abundance ratio (x-axis, Log_2_ (+/−)) and corresponding adjusted *p* value (−Log_10_ (+/−)). Peptide groups in the (+) group that met the significance cutoff (abundance ratio was ≥ 1 and adj. *p* value ≥ 1.301029996) are signified by the *green box*. Groups that hit the maximum cutoff values (outlined in a *gray dashed line*) and that overlap are outlined within the bracket. *C*, STRING (110) analysis of proteins identified in *B* grouped by k-means clustering. The Reactome Pathways analysis of each group is highlighted in color. *D*, classical logo representation of Mast2's consensus sequence. All the PSMs for each significant peptide group identified in *B* were aligned along the defined modification site and imported into WebLogo (v3.7.12). Basic residues are highlighted in *blue*, polar in *green*, acidic in *red*, neutral in *purple*, and hydrophobic in *black*. *E,* differential gene analysis of significant (+) upregulated proteins identified in *B* was entered into the Enrichr gene set enrichment analysis database (107, 108). Results from both WikiPathways (2024) Human and GO Biological Process (2025) were aligned vertically and stratified by combined score. Graphics were created in BioRender. Lemke, M. (2025) https://BioRender.com/u99b092. MAST, microtubule-associated serine/threonine; SIKALIP, stable-isotope kinase-assay-linked phosphoproteomic; LC-MS/MS, liquid chromatography tandem mass spectrometry; PSM, peptide spectrum match; FSBA, 5-(4-fluorosulfonylbenzoyl) adenosine; GO, Gene Ontology.

To identify putative downstream target proteins, we used purified Mast2 (294–1197) in a stable-isotope kinase-assay-linked phosphoproteomic screen (20, 35) (Fig. 8*A*) Using this method, we identified Mast2 (294–1197) substrates with [^18^O]-ATP in human protein lysates. The isotopically heavy phosphate (^18^O) modification labeled 156 total peptide groups, with 59 unique to the exogenous Mast2 (+) samples. There were 30 significantly upregulated proteins, including Mast2 itself (Fig. 8*B*). Due to the binary nature of the assay, the highest enriched peptide groups reached the maximum abundance ratio cutoff, as they did not appear in the (−) control. The gene names corresponding to the peptide groups were entered into the STRING database and demonstrated Reactome pathway enrichment of two groups corresponding to the regulation of the mRNA processing and signaling by Rho-GTPases (Fig. 8*C*). Aligning peptides that matched back to a peptide group around their ^18^O modification, showed that Mast2 had a slight preference for N-terminal acidic, polar, and hydrophobic residues, and C-terminal polar and basic residues (Fig. 8*D*). MASTL also has been shown to prefer basophilic and polar phosphosite locations (20, 22, 47). Pathway analysis of the corresponding gene list using the Enrichr database, with Gene Ontology (GO) biological processes (2025) and WikiPathways (2024), demonstrated enrichment in the control of gene expression, chromatin state, RNA metabolism, organelle and cytoskeletal remodeling, and disease-related signaling pathways (Fig. 8*E*).

**Figure 9:**
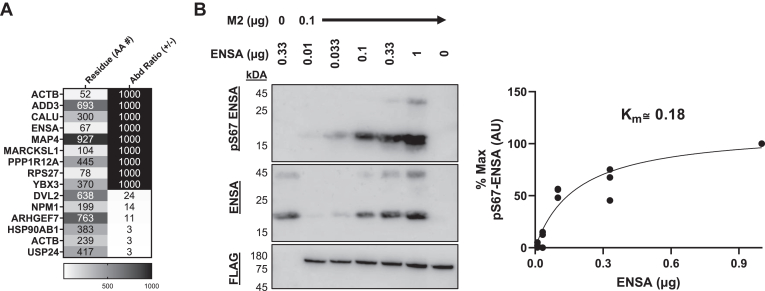
**Mast2 (294–1197) robustly phosphorylates S67-ENSA.***A,* the site-specific information of selected significantly upregulated peptides from Fig. 8*B* (*green box*). *B,* nonradiolabeled substrate assay of ENSA (MCE) phosphorylated by Mast2 (294–1197). Total ENSA and FLAG immunoblot are shown with a representative pS67-ENSA blot (*right*), and phosphorylation was quantified by percent maximal AU with each aligned data point shown over a nonlinear curve determination (*left*). K_m_ was calculated using GraphPad Prism. MAST, microtubule-associated serine/threonine; ENSA, endosulfine-α.

We aimed to validate a Mast2 potential substrate using a parallel method to the substrate screen. Serine 67 (S67) in ENSA was one of the most upregulated phosphosites (Fig. 9*A*). This site was selected for further investigation as reliable and confirmed high-quality antibodies were available (Cell Signaling 5240S and 8770S), and S67 is a well-characterized target site for MASTL (14, 22, 23, 34). MASTL phosphorylates ENSA at S67, activating ENSA and thereby inhibiting PP2A (14). Functional overlap in substrates between MAST kinases has been previously suggested (18, 48). MAST3 activates cAMP-regulated phosphoprotein of 16 kDa (ARPP-16), which is a splice variant of ARPP-19 (49). ARPP-16/19 are highly homologous to ENSA, conserving the S67 site (S46 in ARPP-16, S62 in ARPP-19), but are shorter at the N terminus (49).

Using ENSA as a substrate, we observe that Mast2 (294–1197) shows high affinity for S67-ENSA phosphorylation (K_m_ ≈ 0.18 μg, Fig. 9*B*). Using the predictive capabilities of AlphaFold 3, we also predicted the interaction between whole sequences of MAST2 (Q6P0Q8) and ENSA (O43768), and MAST2 with pS67 ENSA. The binding interface of ENSA/pS67 ENSA was directly adjacent to the catalytic aspartate (RD pocket) around the T-loop (Fig. 9*C*). Phosphorylated S67 ENSA was well packed into the active site of MAST2, partially directed by hydrophobic interactions in the latter half of the T-loop (Fig. 9*C*). Our data collectively identify ENSA as a novel and robust substrate for Mast2.

## Discussion

MAST1-4 are AGC kinases based on sequence homology within the kinase domain. However, the long, intrinsically disordered tri-domain architecture, along with atypical activation segments and C-terminal tails, makes them unique within the AGC family. Our data collectively identified several key features of Mast2 biochemistry that serve as a foundation for understanding MAST kinase biology. We show that the DUF domain and key MAST-conserved residues in the T-loop and C-terminal tail are required for *in vitro* activity on an artificial substrate. Mutational analysis of the T-loop of Mast2 kinases supports the conclusion that MAST kinases do not follow the current model of AGC kinase phospho-activation. Like other AGC kinases, insulin stimulates Mast2 phosphorylation and activity in an mTOR-dependent manner, potentially *via* Mast2's HM or unique HLM. We also demonstrate that Mast2 can phosphorylate ENSA, like MASTL or MAST3 phosphorylation of ARPP-16, supporting the conclusion that there is a functional overlap among MAST kinases.

Using *in vitro* phosphorylation and ligand interactions, the topology that defines the active conformation has been captured for several AGC kinases, including PKC (Protein Data Bank [PDB]: 2I0E) and AKT (1O6L). While the structures of MAST1-4 kinase domains are not solved, MASTL without the T-loop insertion has been solved (5LOH and 8V5H). The PDZ domains of MAST1-4 (3PS4, 2KQF/2KYL, 3KHF, and 2W7R), as well as the DUF domain from MAST1 and MAST3 (2M9X, 1V9V) have been solved. We found that Mast2 (294–867) ΔPDZ had very similar kinase activity to Mast2 (294–1197) (Fig. 4*B*), suggesting that the PDZ domain is not required for activity on artificial substrate. Interestingly, studies in flies have shown that all three *dop* domains must be intact for *in vivo* functionality (50). Several studies have reported an interaction between the PDZ domain of MAST kinases and proteins such as USP1, SNTB2, PTEN, RABV-G, NHE3, and ACE2, which are involved in diverse processes like longevity, neurite outgrowth, and viral infection (4, 51, 52, 53, 54). Generally, PDZ domains form homodimers or heterodimers and bind to protein C termini (55). MAST2 has been shown to recognize C-terminal X-^S^/_T_-X-V/I/L (Class I) PDZ binding motifs (4, 51, 56, 57). Of our substrate screen hit proteins, DVL2 has a PDZ domain, and MAP4 was the only protein that had the closest C-terminal PBM at the lowest stringency (Scansite (58), ∼2% percentile). MAST2 has been shown to interact with the unphosphorylated PBM of RSK1 and the SARS-CoV-2 E protein in PDZ interactome screen experiments (59, 60). Altogether, our results and the literature support the notion that the PDZ domain in Mast2 functions similarly to its role in other proteins, facilitating specific substrate interactions *in vivo* that are nonessential for artificial substrates.

In contrast to the PDZ domain, the function of the DUF1908 domain remains largely mysterious (4, 50, 61). The domain likely emerged before the PDZ, around or preceding the MAST/MASTL divergence in ancient eukaryotes (10). The DUF domain consists of an unstructured region at the N terminus and a hydrophobic alpha-helical barrel (alpha barrel hereafter) just before the kinase domain (4, 10, 61). The Mast2 (294–1197) construct was able to tolerate truncation of the unstructured part of the DUF domain (Fig. 1). However, further truncation of the DUF alpha barrel significantly reduces kinase activity and cannot be rescued *in trans* (Fig. 4). Our results could be explained by the predicted interaction in the current structural model of MAST2 (Q6P0Q8), which shows a contact interface between the DUF: T-loop and the paired ion-side chains, along with the latter half of the T-loop insertion (EKDARE), that collectively support the DFG^in^ conformation shown the predicted MAST2 structure (Fig. 5). This DUF: T-loop coordination model is supported by inactivation caused by EKDARE-alanine mutations, which would disrupt predicted ionic interactions.

The molecular role of the DUF domain is unknown, although deletion mutants fail to rescue *dop* lethality (50). Indeed, humans with single-point mutations in the DUF domain, especially within the hydrophobic core of the alpha barrel, are associated with DUF helical destabilization and severe neuronal disabilities (4, 10, 18). The DUF domain is frequently phosphorylated, particularly at the N terminus (4, 46). The role for DUF phosphorylation is unclear, but it may act as a regulatory hub that influences kinase activity through allosteric mechanisms and protein-protein interactions, such as with 14-3-3 proteins (10, 62). The N-terminal DUF sequence also binds TRAF2 and TRAF6, linking MAST kinases and DUF stability to E3 ubiquitin ligase activity and NF-κB/Wnt signaling pathways (63), which was also revealed through our SIKALIP analysis (Fig. 8*E*). The evidence overall supports the importance of the DUF domain in the function of tri-domain MAST kinases. However, the proposed intramolecular allostery should be tested more thoroughly using physical interaction techniques, such as coimmunoprecipitation, proximity ligation, fluorescence resonance energy transfer (FRET), structural methods, or mass spectrometry.

Distally related AGC kinases, such as PKA, PKC, AKT, S6K, RSK, and MSK, are all activated by T-loop phosphorylation (8). MASTL kinase activity is also likely potentiated by multiple phosphorylation events in its T-loop by Cyclin-B-CDK1 (T194, T207, and T741) (20, 24, 27, 64). Some AGC kinases do not operate *via* T-loop phosphorylation. Rho-activated protein kinases (ROCK, DMPK/MRCK), and G protein–coupled receptor kinases (GRK), which localize with the MAST kinases at the base of the AGC superfamily (2), are not stimulated by T-loop phosphorylation and instead operate *via* intermolecular or intramolecular allosteric stimulation (8). The DUF: T-loop interface partially hallmarks the predicted structure of MAST2's activation loop. A possible innate T-loop salt bridge, located in the canonical AGC phosphorylation site of the namesake residue (26, 40), also potentially stabilizes the DFG^in^ conformation. In theory, the bridge would function as a naturally occurring phospho-mimic of the basal T-loop phosphosite. The bulky T-loops of MAST kinases may instead utilize a salt bridge between a positively charged side chain at DFG^+3^ (K658) and a negatively charged side chain at APE^-11^ close to the canonical APE^−9^ phosphothreonine position. Our results from the ion-repulsion mutant K658D suggest the necessity of a positive side chain in the DFG^+3^ position for basic catalytic conversion in Mast2, possibly due to the paired Asp at APE^-11^ (D682) (Fig. 5). However, this does not eliminate the alternative explanation that mutation could alter substrate binding or specificity (25, 65). Our analysis is limited by the structure available to us, which is only a prediction. Methods such as crystallography or NMR are best suited to directly confirm first the presence of the bridge in the domain, as well as its functionality in MAST kinase catalysis or substrate binding.

We show that Mast2 can be stimulated by insulin in an-mTOR dependent manner (Fig. 7). AGC kinases are known to be phosphorylated on the C-terminal tail by either mTOR complex, primarily with mTORC2 at the TOR-interaction motif (TIM) and C-terminal tails of AKT, PKC isoforms, SGK, and PKN (8, 30, 66). The only AGC kinase shown to be targeted by mTORC1 is S6K (30). At least one phosphosite in MAST2, S900, has been previously identified as rapamycin-sensitive, but was not essential for activity (Fig. 6) (33). Inhibition of mTORC1 is enough to diminish insulin stimulation of Mast2 regardless of TM phosphorylation (Fig. 7). Although mTORC2-related phosphorylation cannot be ruled out this way, we can conclude that mTORC1 is likely upstream of Mast2 during anabolic stimulation. The lack of change of TM phosphorylation with Torin1 suggests pS825 is not mTORC2-dependent, as has been presumed for the TMs of PKC and AKT (44). The phosphorylation of AKT's TM by mTORC2 is both cotranslational and nonessential (66, 67). Purified Mast2 from insulin-stimulated cells indicates that mTORC1 phosphorylation probably occurs between the HM or HLM, or at a related site that facilitates autophosphorylation of the HM or HLM, similar to PKC or AKT (66). This is likely the case for MASTL's TM, its sole C-terminal motif, which is probably autophosphorylated at S875 (20, 27). MASTL autophosphorylation is primed after coordinating multiple phosphorylation events by CDK1 (T194, T207, and T741), AKT (T299), and mTORC1 (S878) (20, 28, 34). It has been proposed that the HM of other molecules then facilitates the full activation of MASTL (45). Our data demonstrate the functional importance of TM, HM, and HLM sites, placing Mast2's kinase domain extension potentially downstream of insulin-mTOR signaling, and lays the foundation for further investigations into the conserved but highly irregular C termini of MASTs.

We sought to understand not only the regulation of MAST kinase activity, but also the functional outputs of Mast2. Identified initially as “microtubule-associated” kinases, our proteomic substrate screen identified many cytoskeletal-associated proteins targeted by Mast2 (Figs. 8/9*A*, S2) (68, 69, 70, 71, 72, 73, 74, 75, 76, 77, 78, 79, 80, 81). In flies, *dop* depletion causes decreased dynein light intermediate chain and lethality (50). Plant MAST homologs, such as IREH1, lack the PDZ domain and regulate root-hair elongation, which depends on microtubules and actin cytoskeleton (82, 83). IREH1 also colocalized to centrosomes when expressed in mammalian cells (61). This potentially links to recent evidence that MAST4's kinase domain interacts with the dynein-light chain at the primary cilium (84). Previous mass spectrometry screening experiments have also implicated Mast2 as an interacting protein with dynein light chain LC8 in mouse brains (85). MASTL has been linked to both tubulin and actin cytoskeletons, as MASTL phosphorylation of tubulin has been identified in proteomic screens (20, 22), and MASTL may regulate cell movement and actomyosin contraction independently of kinase activity (86). These hits underscore the conservation of a specific but perhaps indirect cytoskeletal regulatory role, particularly for the actin cytoskeleton; however, this requires further validation.

In addition to novel Mast2 substrates, we also found overlap with previous MASTL screens. ENSA or ARPP16/19, the most studied Mast-kinase substrate, negatively regulates PP2A to promote mitosis (21, 23, 49), is targeted at the conserved DpSG motif (S46 in ARPP-16, S62 in ARPP-19, and S67-ENSA) (24), which was readily phosphorylated by Mast2 (294–1197) in the SIKALIP screen and *in vitro* (Fig. 9). In fact, 14 proteins identified in our Mast2 substrate screen were previously identified with MASTL screens (Fig. S3) (20, 22, 87, 88, 89, 90, 91, 92), including ACTB, PPP1R12A, MAP4, ADD3, CALU, and HSP90AB1 from the cytoskeletal-Mast2 hits (Fig. S2). The most recurring overlapping hit was nucleophosmin-1 (NPM1) where we find that Mast2 targets a site (T199) involved in centrosome duplication, pre-mRNA processing, and DNA damage (87, 89, 92). We also want to highlight Y-box binding protein transcription factor isoforms (YBX1 for MASTL; YBX3 for Mast2) (93). Another notable hit was a small ribosomal subunit protein 27 (RPS27, S78), which belongs to the RPS family and is associated with AGC kinase-mTORC1 signaling (94, 95). The other dominant signal from our substrate screen was regulation of mRNA splicing (Fig. 8), which aligns with cluster analysis of MASTL substrates (22). This could represent the functional output of mTOR-MAST signaling, since mTOR activity stimulates SRSF proteins and growth-related RNA splicing (96, 97). Comparing substrates may indicate overlapping biological niches of MAST kinases that collectively regulate mRNA, cilium, and protein phosphatase dynamics (Fig. S3).

Heat shock protein 90 (HSP90) was a robust Mast2-MASTL target (Fig. S3) and has been implicated in MAST1/MASTL stabilization (88, 90). To this point, MAST2 was initially shown to immunoprecipitate with and phosphorylate an unknown 75 kDa substrate (36), which we possibly identified as HSP70 (Fig. S1). MAST kinase stability is also likely regulated by ubiquitination, as MAST1 was also shown to be protected by ubiquitin-specific peptidases (USPs), USP1/USP28, and provided cisplatin resistance to cancer cells (98, 99). We identified a USP downstream of Mast2 in our screen (USP24, S417). MAST kinases are very large and intrinsically disordered compared to other AGC kinases, and MAST1-4 are all highly associated with binding 14-3-3 proteins (10). Considering this, binding to stabilizing and modular chaperone proteins, such as HSPs and 14-3-3s, as well as deubiquitinase activity by USPs, may be a predominant method of MAST kinase functional assembly, cellular localization, or stability.

In conclusion, our data support the requirement of the DUF domain and key residues in Mast2's activation segment, which does not appear to follow AGC-canonical activation. Mast2 also potentially falls downstream of the mTORC1 anabolic signaling cascade in its C-terminal tail, independent of the TM. Substrate screening identified novel Mast2 and previously identified MASTL/MAST3 targets, suggesting functional overlap on at least one substrate—ENSA. Collectively, our work addresses fundamental questions regarding the biochemical profile of MAST kinases and allows the field to gain a deeper understanding of their physiological role.

## Experimental procedures

### Materials

γ-[P^32^]-ATP was purchased from PerkinElmer Life Sciences. The FLAG beads (A220), peptide, primary FLAG antibody (F1804), and secondary anti-rabbit/anti-mouse-horseradish peroxidase-conjugated antibodies were from Millipore Sigma. Primary antibodies for total AKT (2920S), phospho-specific AKT (pT308, 9275S and pS473, 4060L), phospho-specific S6K (pT389, 9205S), phospho-specific ENSA (pS67, 5240S), and total ENSA (8770S) were from Cell Signaling. Total S6K1 antibodies were previously reported (100). Mast2 phospho-specific S825 (S825) antibodies were generated using Yurogen Biosystems LLC. Briefly, polyclonal antibodies were raised to the TM phosphopeptide (C-HH(Nle)D(p-S)EDEEE). Nle was substituted for Met (human)/Val (mouse) so the antibody would recognize both sequences. Subsequent antiserum was pooled from three rabbits, and the antibody was purified *via* antigen affinity chromatography. Recombinant human N-terminal His-tagged ENSA was purchased from MedChemExpress (MCE; HY-P74175).

### Cell culture

HEK293, HEK293T, and HEK293E were cultured in high-glucose Dulbecco's modified Eagle's medium plus L-glutamine, supplemented with 5% fetal bovine serum (GeminiBio; Benchmark SKU# 100–106–500) and 1% Antibiotic-Antimycotic (Thermo Fisher Scientific #15240062) as previously described (101). Serum starvation media generated for insulin stimulation experiments were supplemented with 2.5% bovine serum albumin (BSA), 0.25% fetal bovine serum, and 1% antibiotic-antimycotic.

### Expression vectors

Sequence design, DNA digests, PCR, ligations, and other recombinant DNA procedures were performed using standard protocols. Subsequently, plasmid vectors containing recombinant proteins were sequenced to confirm the correct protein of interest insertions. When referring to amino acid numbering, unless otherwise indicated by Uniprot accession code, we are referencing *Mus musculus* Mast1 (1570 amino acids, NP_064329.2/Q9Y2H9), *M. musculus* Mast2 isoform 2 (1794 amino acids, NP_032667.2/B1AST8), *M. musculus* Mast3 isoform 5 (1305 amino acids, NP_955012.2/A0A1D5RM97) and *Homo sapiens* Mast4 isoform 3 (2623 amino acids, NP_001158136.1/O15021). For brevity, all point mutations/motifs highlighted will be numbered by their position in Human Mast2 (Q60592). The full-length Mast2 was a generous gift from Chris Yun and inserted into pFLAG-CMV4 (36, 53). All other Mast2 constructs were generated by either PCR mutagenesis and placed into a pcDNA3-FLAG/pLV-Puro-CMV-FLAG-Twin-Strep-tag (IBA) vector or were purchased directly from Vector Builder. Mast1 (1–1063) was placed into a pcDNA3-FLAG vector, and Mast3 (1–1043) was put into a pcDNA3-FLAG-Twin-Strep-myc expression vector. Human MAST4 (173–1363) was synthesized (GENEWIZ) and inserted into a pcDNA3-FLAG vector.

### Recombinant proteins

Subsequently, 15-cm plates of HEK293T or HEK293E cells were transfected with 15 to 30 μg of tagged plasmid expression vectors described above, using a 2:1 or 3:1 ratio of polyethyleneimine: DNA, and then fed for 48 to 72 h. Multiple independent preparations of recombinant proteins were generated by washing the cells with ice-cold phosphate-buffered saline and subsequent Potter-Elvehjem homogenization in Buffer A (1 mM EDTA, 1 mM EGTA, 1 mM DTT, 0.1% Tween 20, 10 mM sodium phosphate, and 50 mM β-glycerophosphate, pH 7.4, containing protease inhibitors (10 μg/μl leupeptin and pepstatin, and 1 mM PMSF)) (102). Lysates were centrifuged at 16,000*g* for 10 min at 4 °C, and the supernatant was incubated either with compacted FLAG (M2) beads at 15 μl volume/plate or 200 μl of compacted Strep-Tactin XT beads (IBA) for 2 h with rotation at 4 °C (103). The beads were loaded onto a separation column and washed three times with buffer A + 0.1% Tween-20. Bound proteins were eluted with two volumes of 1 mg/ml FLAG peptide in buffer A without Tween-20 or protease inhibitors or buffer BXT with 50 mM Biotin without detergent or protease inhibitors. The protein purifications were loaded on an SDS-polyacrylamide gel along with ascending amounts of BSA standard of known concentrations. Proteins were stained with Coomassie brilliant blue, and the gels were imaged using an Amersham ImageQuant 800 (iQ800) imaging system and quantified using ImageQuant TL.

### *In vitro* kinase assays

Unless otherwise noted in figure legends, recombinant proteins, as generated *via* the prior section, were incubated with a solution containing 10 μg of MBP and 200 μM ATP supplemented with ≈ 7500 to 12000 cpm/μM or 2 to 3 μCi ATP of [γ-^32^P]-ATP, 0.25 μM Microcystin, 2 mM MgCl_2_, and 1 mM DTT in kinase buffer B (50 mM NaCl_2_, 0.1 mM EGTA, 5 mM Hepes, and 50 mM β-GP) for 30 min at 30 °C with agitation. Assays were quenched by adding 5× Laemmli sample buffer and then heat-shocked at 75 °C for 5 min before resolving the reaction using an SDS-PAGE gel. The proteins were then transferred onto a polyvinylidene fluoride (PVDF) membrane, exposed to either an autoradiographic film or a phospho-intensifying screen (GE Healthcare), and subsequently imaged using a Typhoon biomolecular imager (GE Healthcare).

### Western blot analysis

Protein expression was verified *via* SDS-PAGE. PVDF membranes were blocked in Tris-buffered saline with Tween-20 (TBST; 150 mM NaCl, 0.05% (w/v) Tween-20, and 50 mM Tris, pH 7.4) with 10% (w/v) dried milk for 1 h at room temperature. Subsequent primary antibodies were incubated at a 1:1000 dilution in TBST buffer containing 5% BSA and 0.02% sodium azide for 1 h at room temperature. Horseradish peroxidase-conjugated secondary antibodies were diluted 1:10,000 in 2% (w/v) dry milk or 5% BSA for 1 h at room temperature. The PVDF membranes were incubated with TBST before exposure to the chemiluminescent substrate (SuperSignal West Pico PLUS, Thermo Fisher Scientific) and then imaged using the iQ800.

### Sequence and structural analysis

Mast kinase protein sequences were acquired from Uniprot or NCBI (hMAST1-Q9Y2H9; mMast1-Q9R1L5; hMAST2-Q6P0Q8; bMAST2-E1B754; mMast2-B1AST8; zmast2-E7F723; B3S1R6; do*p-*Q9VUQ9; hMAST3-O60307; mMast3-A0A1D5RM97; hMAST4-O15021; mMast4-Q811L6; hAKT1-P31749; hS6KA1-Q15418; hPKCB2-P05771–2; hENSA-O43768; hHSP70-P0DMV9). Percent similarity was determined by positive designations from the protein basic local alignment search tool (NCBI-pBLAST); identical residues aligned by pBLAST defined the percent identity. Multiple sequence alignment, degree of conservation, and phylogeny branch length scores were performed using EMBL's Clustal Omega tool and visualized in iTOL (104). Modeling and visualization of structures were superimposed in Pymol (RMSD generated in Pymol during superposition). Predictions of MAST2 + HSP70, MAST2 K658D, and MAST2 + ENSA/pS67 ENSA were generated in AlphaFold 3 (105). Hydrophobicity is colored by the normalized consensus hydrophobicity scale as described previously (Color h, (106)).

### SIKALIP screen

The stable isotope-labeled kinase assay-linked phosphoproteomic experimental design was modified from previous uses of the method (20, 35). Briefly, two 15 cm plates of nontransfected HEK293T cells were lysed using Potter–Elvehjem homogenization in 2 ml total of SIKALIP lysis buffer (50 mM Tris–HCl, pH 7.5, 150 mM NaCl, and 5 mM EDTA). The lysate was centrifuged at 16,000*g* for 10 min at 4 °C. Then 400 μg of total protein from the supernatant was diluted to 200 μl final volume. The lysate was treated with 1 mM 5-(4-fluorosulfonylbenzoyl) adenosine (FSBA), dissolved in 10% dimethyl sulfoxide, for 1 h at 30 °C. Treated lysates were then immediately incubated in 10 U of FastAP phosphatase and 10x rAPid phosphatase buffer for 1 h at 37 °C (Thermo Fisher Scientific). The samples were then heat shocked at 75 °C for 5 min. Unreacted FSBA was removed with a 30 kDa filter (Sartorius Vivaspin 500). Samples were washed on the column with 200 μl of lysis buffer three times and then concentrated to 200 μl. Kinase reactions then occurred on the filter with the addition of 400 ng of murine FLAG-Mast2 (294–1197) in the presence of 1 mM [γ-^18^O_4_]-ATP (Cambridge Isotope Laboratory) and 10 mM MgCl_2_ for 1 h under agitation at 30 °C. Reactions were quenched by adding 8 M Urea and 5 mM DTT and heat-inactivated at 65 °C for 15 min.

### Phosphoproteomics sample preparation

Quenched SIKALIP samples underwent buffer exchange to 200 μl 6 M urea: 25 mM ammonium bicarbonate on a 10 KDa filter (Millipore). Samples were alkylated with 50 mM iodoacetamide at room temperature for 30 min, followed by buffer exchange to 200 μl 25 mM ammonium bicarbonate. Samples were digested to peptides using trypsin/Lys-C (5 μg in 10 μl of 25 mM ammonium bicarbonate, sequencing grade from Promega), which was added to the mixture and incubated at 37 °C for 16 h. Digested peptides were collected through the filter (14,000*g*, 15 min) and lyophilized by speed-vac. Phospho-samples were further enriched by TiO2 phosphopeptide enrichment kit (Thermo Fisher Scientific), and the final elution buffer was dried out through speed-vac. Phosphopeptides were suspended in 25 μl buffer A (0.1% formic acid) and processed for LC-MS/MS analysis.

### LC-MS/MS analysis

Nano-electrospray ionization-LC-MS/MS analyses were performed using an Easy-nLC 1200-Orbitrap Q Exactive Plus mass spectrometer (Thermo Fisher Scientific). The peptides were eluted from the trap column and through a homemade nanocapillary analytical column (20 cm, 5 μm C18 packed in 360 μm o.d. × 75 μm i.d. fused silica), with an integrated electrospray tip, using a 180 min 1 to 95% reverse-phase LC gradient (A: 0.1% formic acid; B: 80% acetonitrile, and 0.1% formic acid) with the following parameters: 0 to 1.48 min 1% B, 400 nl/min; 1.48 to 2:00 min 1% B, 300 nl/min; 2 to 90 min 16% B; 90 to 146 25% B; 146 to 147 min 95% B; 147 to 153 min 95% B; 153 to 154 min 1% B; 154.0 to 154.1 min 1% B, 400 nl/min; and 154.1 to 180 min 1% B, 400 nl/min. A top ten data-dependent acquisition MS method was used.

### Phosphoproteomics data analysis

The phosphopeptide identification and quantification pipeline was processed on the Proteome Discoverer (Thermo Fisher Scientific) with an internal third-party Byonic search engine. The reference database used UniProt human proteomes with manually added mouse MAST2. Processing parameters included trypsin (full) as enzyme, 10 ppm precursor mass tolerance, 20 ppm fragment mass tolerance, 1% protein false discovery rate, variable (common) modification of phosphorylated amino acids (+79.966331 Da for S, T, Y) and ^18^O_3_-replaced phosphorylated amino acids (+85.97907 Da for S, T, Y), and fixed modification (+57.021465 Da for alkylated cysteine residues). Consensus steps included peptide spectrum match (PSM) filters of 5 ppm delta mass and Byonic score ≥ 200, total peptide amount for normalization, and *t* test for hypothesis test. Ratios were calculated pairwise ratio-based. The result was presented as peptide-abundance-ratio of samples with exogenous mouse MAST2 over samples with absent mouse MAST2. The abundance ratios' maximum and minimum values were set as default (1000 *versus* 0.001) before the log_2_ calculation. Data were pooled from two independent experimental preparations with or without exogenous Mast2. Peptide groups were exported from the Proteome Discoverer data pipeline (v3.1.0.638, Thermo Fisher Scientific) using the Byonic search engine. UniProt accession numbers were imported into the DAVID gene ID conversion tool to acquire master gene IDs. The maximum abundance enrichment was set to ≥ 9.965784285 (log_2_) with corresponding adj. *p*-value 16.06789306 (log_10_). Upregulated groups were considered significant (green) if both the log_2_ abundance ratio was ≥ 1 and the -log_10_ adj. *p*-value ≥ 1.301029996 (0.05). All Ser/Thr modified peptide groups were entered into the STRING interaction database, clustered into two groups by k-means clustering, and analyzed by the Reactome Pathways database. Significant PSMs with a defined S/T O^18^ modification were aligned along their modification site and imported into WebLogo (v3.7.12). Multiple PSMs containing O^18^ modifications in matched peptide groups were entered into the logo, equal to the number of PSMs that matched back to the group. Differential gene analysis was conducted *via* the Enrichr database (107, 108, 109) and stratified by combined score (log (*p* value) ∗expected rank z-score deviation). The mass spectrometry proteomics data have been deposited to the ProteomeXchange Consortium *via* the PRIDE partner repository with the dataset identifier PXD069171 and 10.6019/PXD069171.

### Statistical analysis

All statistical analysis of the results was performed in GraphPad Prism software (10.2.2). Root-mean-squared deviation calculations of superimposed structures were performed in PyMol. Nonlinear regression of the Michaelis–Menten calculations was used to determine K_m_ and V_max_. Generally, significance between two groups was determined *via* unpaired *t*-testing, and for more than two groups *via* one-way/two-way analysis of variance (ANOVA) followed by *post hoc* testing. Replicate values are shown and/or represented as mean determinations ± standard deviation of the mean (SD).

## Data availability

The mass spectrometry proteomics data for this study have been deposited to the ProteomeXchange Consortium *via* the PRIDE (PMID: 39494541) partner repository with the dataset identifier PXD069171 and 10.6019/PXD069171. All other datasets supporting the findings of this study are available within the article and its supplementary information files.

## Supporting information

This article contains supporting information (20, 21, 22,91,105,107,108,110)

## Conflict of interest

The authors declare that they have no conflicts of interest with the contents of this article.
