# Supplementary material for: Characterization of Mast2 kinase defines structural features, regulation, and substrates
Source: J Biol Chem. 2025 Nov 17;301(12):110922. doi: 10.1016/j.jbc.2025.110922 (PMC12732324; doi:10.1016/j.jbc.2025.110922)
Supplement: Figure S1 [file mmc1.pdf]

Supp. Fig. 1) A, Mast2 (294-1197) 70 kDa band excision Mass Spec. Purified Mast2 (294-1197) as per the recombinant FLAG protocol outlined in previous sections was resolved on SDS-PAGE. The band corresponding to the co-purified 70 kDa protein was excised by traditional methods and analyzed using band excision LC-MS/MS by the UVA-Biomolecular analysis core standard protocol. Briefly, gel pieces from the band were loaded into a siliconized tube and washed with 200 µL 50% methanol. The sample was dehydrated in acetonitrile and subsequently rehydrated in 30 µL of 10 mM dithiothreitol in 0.1 M ammonium bicarbonate and reduced at room temperature for 30 min. The DTT solution was removed, and the sample was then alkylated in 30 µL 50 mM iodoacetamide in 0.1 M ammonium bicarbonate at room temperature for 30 min. The reagent was removed, and the sample was dehydrated in 100 µL acetonitrile. The acetonitrile was removed, and the sample was rehydrated in 100 µL 0.1 M ammonium bicarbonate. The sample was dehydrated in 100 µL acetonitrile again, and once the acetonitrile was removed, the sample was entirely dried by vacuum centrifugation. The gel pieces were finally rehydrated in 20 ng/µL trypsin in 50 mM ammonium bicarbonate on ice for 30 min. Any excess enzyme solution was removed, and 20 µL 50 mM ammonium bicarbonate was added. The sample was digested overnight at 37 oC, and the peptides were extracted from the polyacrylamide in a 100 µL aliquot of 50% acetonitrile/5% formic acid. This extract was evaporated to 20µL for MS analysis spectra (Top10 HCD) to determine the amino acid sequence in sequential scans. This mode of analysis produces approximately 25000 MS/MS spectra of ions ranging in abundance over several orders of magnitude. Not all MS/MS spectra are derived from peptides. The data were analyzed by database searching using the Sequest search algorithm against Uniprot Human and Mouse. Results reflect Normalized Total Spectra >10 on the Y-axis and Rank(Total Spectra x average number of spectra for all samples) on the X-axis. B, Whole protein sequences for MAST2 (Q6P0Q8) and HSP70 (P0DMV9) were entered into the AlphaFold 3 webserver (right (105)). A zoomed-in view of the putative interaction site between MAST2 and HSP70 is shown (left). Numbers indicate amino acids. Graphics were created in BioRender. Lemke, M. (2025) https://BioRender.com/38spi2k.

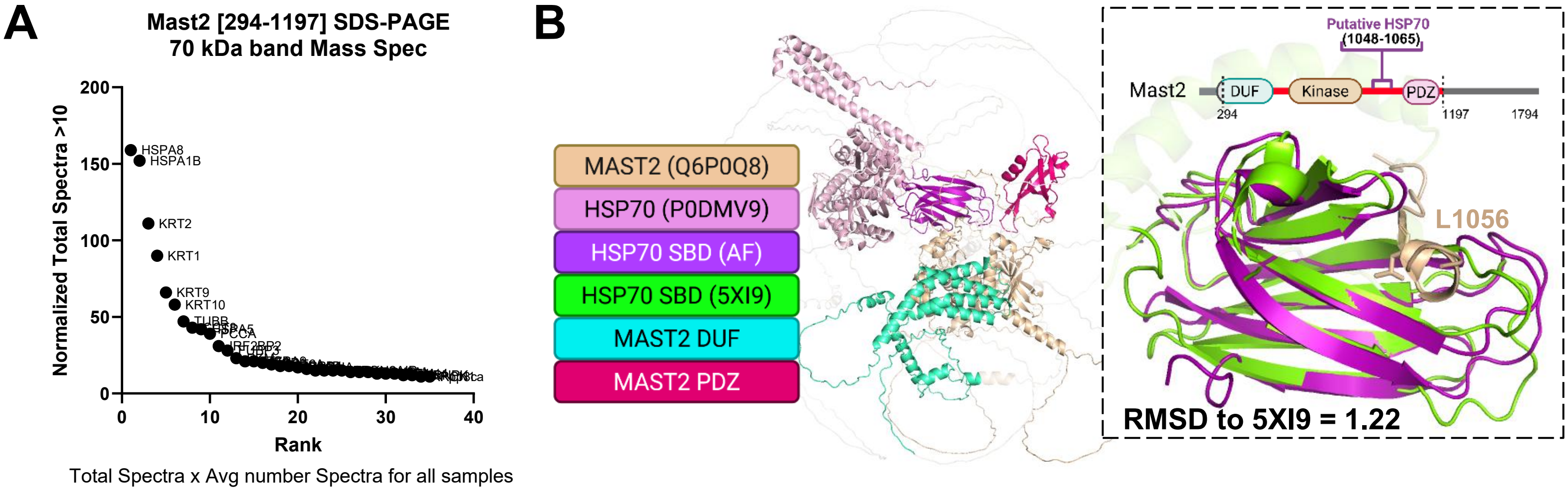
A
Mast2 [294-1197] SDS-PAGE
70 kDa band Mass Spec
Normalized Total Spectra >10
Rank
HSPA8
HSPA1B
KRT2
KRT1
KRT9
KRT10
Total Spectra x Avg number Spectra for all samples
B
MAST2 (Q6P0Q8)
HSP70 (P0DMV9)
HSP70 SBD (AF)
HSP70 SBD (5XI9)
MAST2 DUF
MAST2 PDZ
Putative HSP70
(1048-1065)
Mast2
DUF
Kinase
PDZ
294
1197
1794
L1056
RMSD to 5XI9 = 1.22
