# Supplementary material for: Characterization of Mast2 kinase defines structural features, regulation, and substrates
Source: J Biol Chem. 2025 Nov 17;301(12):110922. doi: 10.1016/j.jbc.2025.110922 (PMC12732324; doi:10.1016/j.jbc.2025.110922)

Supp. Fig 2) Mast2 (294-1197) regulates cytoskeletal proteins. A, Previously identified cytoskeletal function of a selected group of Mast2 SIKALIP targets (Fig. 8) with corresponding PubMed Identification number (PMID). B, The proteins were then entered into the Enrichr gene set enrichment analysis database (107, 108). Results from both GO Biological Process (2025) and Compartments (2025) were aligned vertically and stratified by combined score. C, STRING (110) analysis of proteins identified in A grouped by k-means clustering. The KEGG pathway analysis results are highlighted in color.

A

Name	Protein	Cytoskeletal function	Source PMID
B-actin	ACTB	B-actin- maintaining cell structure and enabling cell motility; pS239 incudes polymerization and increased cell motility	37648284
Protein phosphatase 1 regulatory subunit 12A	PPP1R12A/ MYPT1	Regulates actin-myosin contraction and targets many microtubule-associated substrates; pS445 induces 14-3-3 binding	37810667, 28737169
Microtubule-associated protein 4	MAP4	Phosphorylation of T927 causes dissociation from microtubules during mitosis	31253867
Macrophage myristoylated alanine-rich C kinase substrate like 1	MARCKSL1	Regulates actin dynamics in the brain and cancer	22751924
Γ-adducin	ADD3	PKC substrate; C-terminal MARCKS-related motif that blocks binding to calmodulin, spectrin, and filamentous actin when phosphorylated	12743105, 3511042, 9679146, 31548578
Calumenin	CALU	C-terminal site downstream of the EF-hand domain; secreted protein decrease actin fragmentation and regulate cell cycle	16691550
Disheveled 2	DVL2	Has a PDZ domain; central adapter protein in Wingless and Int-1 (Wnt) signaling that is well known to colocalize and regulate actin through non-canonical Wnt signaling	38877839, 10871283
Rho guanine nucleotide exchange factor 7	ARHGEF7/ β-PIX	Promotes actin polymerization and microtubule remodeling	27012601, 21249427
Heat-shock protein 90 beta	HSP90AB1	Localizes with both actin and microtubules; required for polarization of neurons	24286867

B

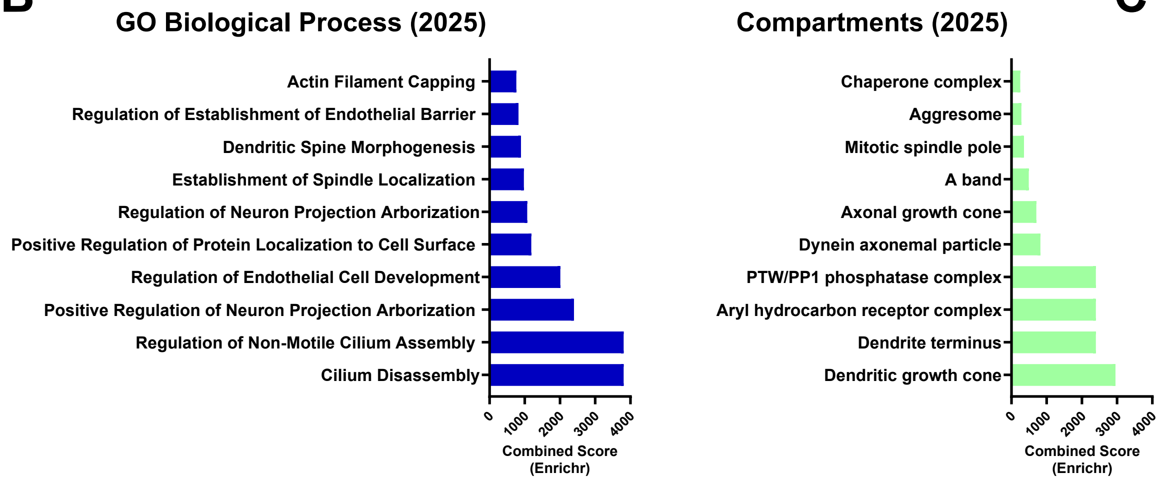

C

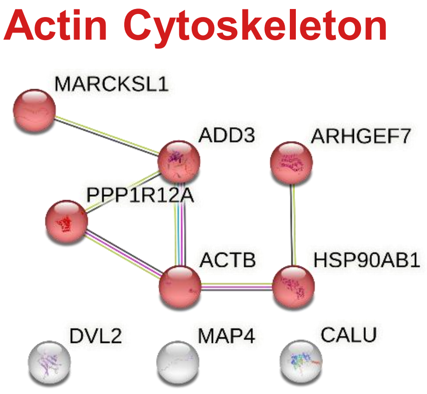

Supplement: Figure S2 [file mmc2.pdf]
