# Supplementary material for: Characterization of Mast2 kinase defines structural features, regulation, and substrates
Source: J Biol Chem. 2025 Nov 17;301(12):110922. doi: 10.1016/j.jbc.2025.110922 (PMC12732324; doi:10.1016/j.jbc.2025.110922)
Supplement: Figure S3 [file mmc3.pdf]

Supp. Fig 3) Mast2 and MASTL demonstrate functional overlap. A, Mast2 SIKALIP (M2-SIKALIP) results (Fig. 8) were compared to published MASTL results (Rogers et al., 2016—ML-IP (91); Hermida et al., 2020—MASTL-SIKALIP (20); Marzec et al., 2022—ML-SILAC (22)). Overlapping results are stratified by the number of screens that identified that protein. B, STRING (110) analysis of proteins identified in A colored by Cellular Component (Gene Ontology)—red—intracellular organelle lumen; blue—nuclear lumen. C, The 14 overlapping proteins were entered into the Enrichr gene set enrichment analysis database (107, 108). Results from both GO Biological Process and Molecular Function (2025) were aligned vertically and stratified by combined score. Graphics were created in BioRender. Lemke, M. (2025) https://BioRender.com/dtfayo9.

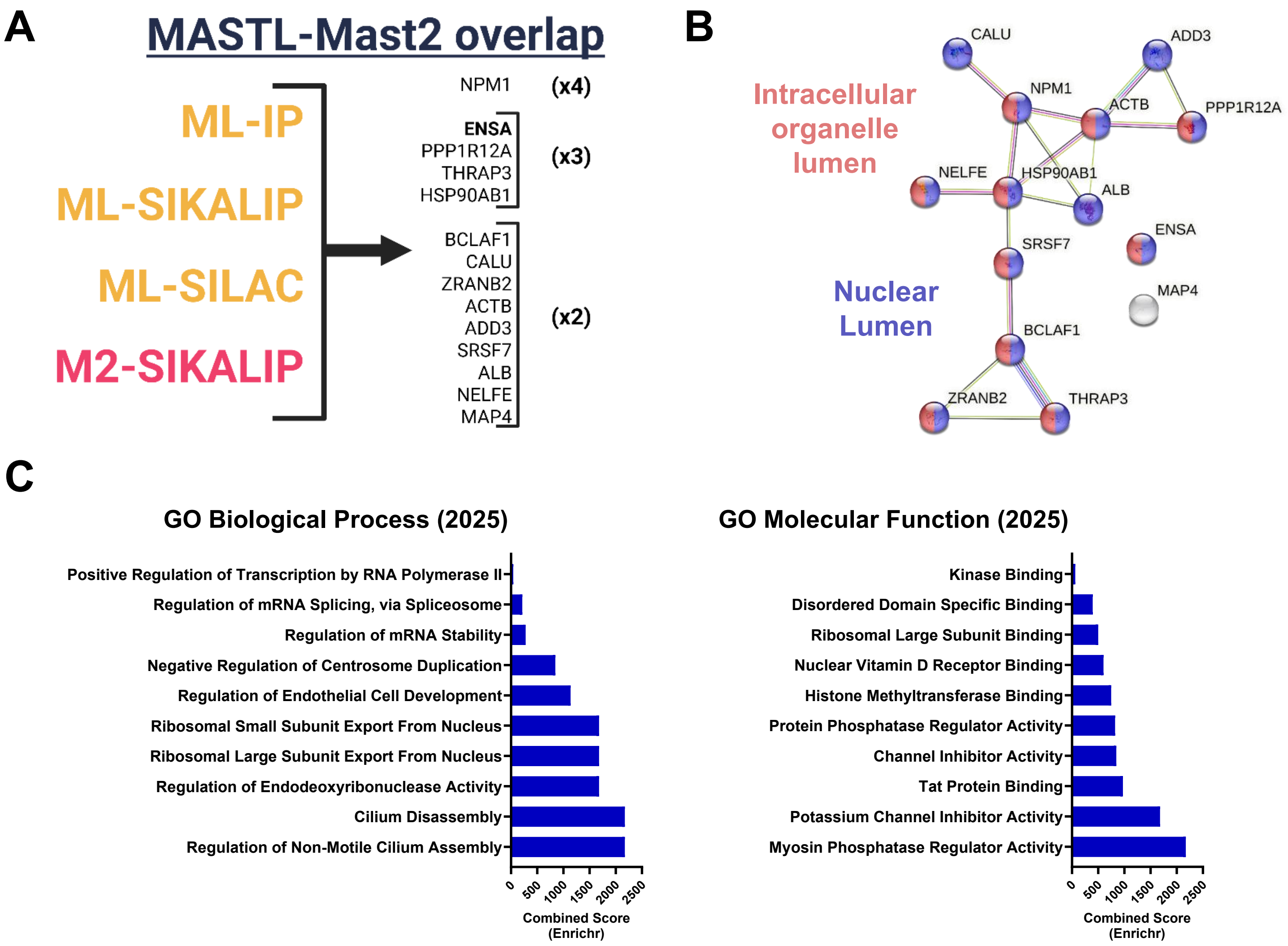
A
MASTL-Mast2 overlap
ML-IP
ML-SIKALIP
ML-SILAC
M2-SIKALIP
NPM1 (x4)
ENSA
PPP1R12A
THRAP3
HSP90AB1
(x3)
BCLAF1
CALU
ZRANB2
ACTB
ADD3
SRSF7
ALB
NELFE
MAP4
(x2)
B
Intracellular organelle lumen
Nuclear Lumen
CALU
ADD3
NPM1
ACTB
PPP1R12A
NELFE
HSP90AB1
ALB
ENSA
SRSF7
MAP4
BCLAF1
ZRANB2
THRAP3
C
GO Biological Process (2025)
Positive Regulation of Transcription by RNA Polymerase II
Regulation of mRNA Splicing, via Spliceosome
Regulation of mRNA Stability
Negative Regulation of Centrosome Duplication
Regulation of Endothelial Cell Development
Ribosomal Small Subunit Export From Nucleus
Ribosomal Large Subunit Export From Nucleus
Regulation of Endodeoxyribonuclease Activity
Cilium Disassembly
Regulation of Non-Motile Cilium Assembly
0 500 1000 1500 2000 2500
Combined Score (Enrichr)
GO Molecular Function (2025)
Kinase Binding
Disordered Domain Specific Binding
Ribosomal Large Subunit Binding
Nuclear Vitamin D Receptor Binding
Histone Methyltransferase Binding
Protein Phosphatase Regulator Activity
Channel Inhibitor Activity
Tat Protein Binding
Potassium Channel Inhibitor Activity
Myosin Phosphatase Regulator Activity
0 500 1000 1500 2000 2500
Combined Score (Enrichr)
